# Supplementary material for: Genetic Profiles and Risk Stratification in Adult De Novo Acute Myeloid Leukaemia in Relation to Age, Gender, and Ethnicity: A Study from Malaysia
Source: Int J Mol Sci. 2021 Dec 27;23(1):258. doi: 10.3390/ijms23010258 (PMC8745150; doi:10.3390/ijms23010258)
Supplement: Supplementary file 1 [file ijms-23-00258-s001.zip › Supplementary S1.pdf]

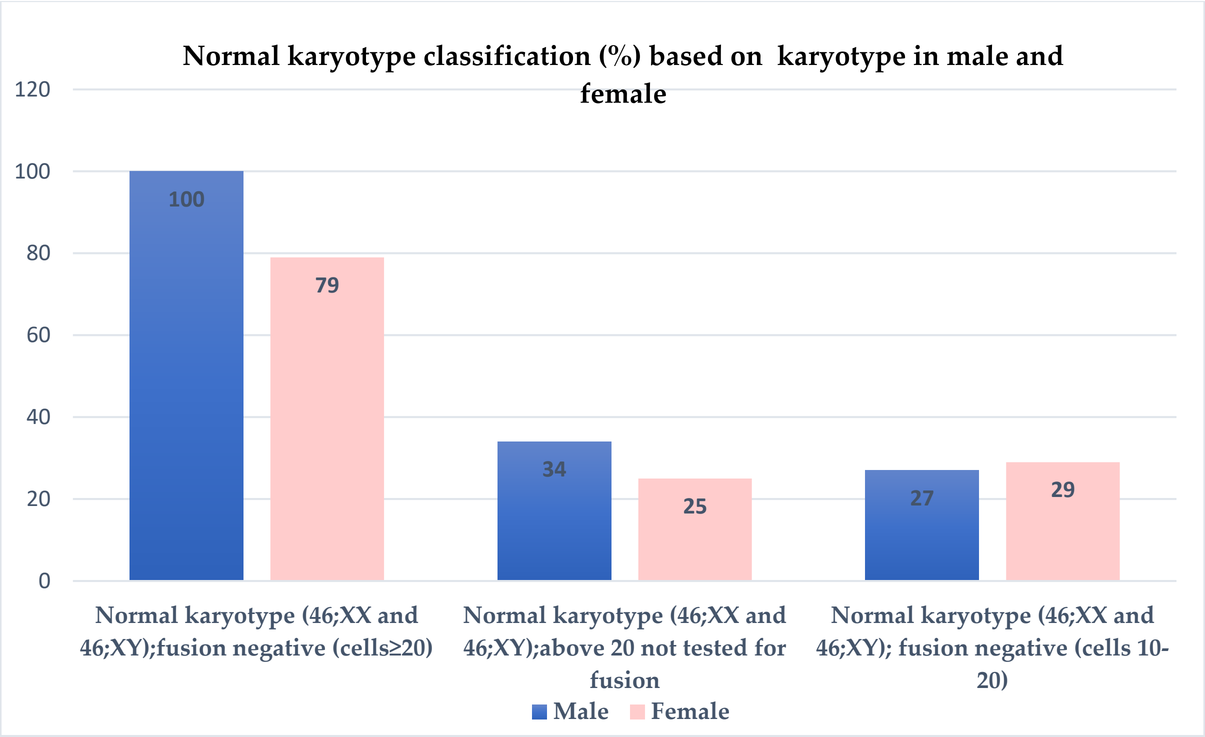

Figure S1. Assignment of AML-normal karyotype based on the metaphase chromosomes and QuanDx fusion findings

Table S1. Summary of assignment of patients into groups based on cytogenetics and molecular genetics (QuanDx fusion kit findings)

Abnormality	t(9;22)	t(15:17)	t(8;21)	inv(16)	MLL karyotypes	t(16;21)	NK[a] (>20 cells)	NK[a] (10-20 cells)	Failed/no results	del(5q)	del(7q)	del(9q)	(+)8	(+)21	(+)22
BCR-ABL	3	1	0	0	0	0	0	0	0	0	0	0	0	0	0
PML-RARA	0	62	0	0	0	0	4	1	8	0	0	0	0	0	0
RUNX1-RUNX1T1	0	0	40	0	0	0	3	0	7	0	0	0	0	0	0
CBFB-MYHIIA	0	0	0	16	0	0	7	2	3	0	0	0	0	0	0
MLL	0	0	0	0	6	0	1	0	0	0	0	0	0	0	0
FUS-ERG	0	0	0	0	0	1	0	0	0	0	0	0	0	0	0

[a] NK: Normal karyotype

Table S2. Fusions and mutations detected in this AML cohort*

	FLT3-ITD+NPM1^{wt}/FLT3-ITD-NPM1+/FLT3-ITD+NPM1+ n=414	PML-RARA n=341	RUNXI-RUNXITI n=459	CBFβ-MYH Ii n=432	BCR-ABL n=279	MLL-MLL T3 n=170	MLL-MLLT4 n=167	MLL-ELL n=166	FUS-ERG n=167
Detected	138(33)	75(22)	50(11)	28(6)	4(1)	3(2)	1(1)	1(1)	1(1)
Not Detected	276(67)	266(78)	409(89)	404(94)	275(99)	167(98)	166(99)	165(99)	166(99)

*Note that although 854 patients were recruited in this study, not all patients were tested for the mutations listed above. Refer to the n value below each mutation

Table S3. Cytogenetic findings, NPM1 mutation, and FLT3-ITD distribution*

	Normal karyotype	t(15;17)	t(8,21)	t(11q23)	inv(16)	del(5q/7q)	Misc Deletion	Trisomi 8	Misc Trisomies	Misc Translocation	Total
**NPM1 mutation**											
Detected	64(88)	2(3)	0(0)	0(0)	0(0)	0(0)	3(4)	0(0)	1(1)	3(4)	73(100)
Not Detected	124(45)	44(16)	25(9)	10(4)	13(5)	14(5)	9(3)	12(4)	8(3)	16(6)	275(100)
Not tested	106(42)	40(16)	26(10)	5(2)	17(7)	18(7)	16(6)	7(3)	5(2)	13(5)	253(100)
Total	294(49)	86(14)	51(8)	15(2)	30(5)	32(5)	28(5)	19(3)	14(2)	32(5)	601(100)
**FLT3-ITD mutation**											
Detected	42(61)	12(17)	3(4)	0(0)	0(0)	1(1)	1(1)	6(9)	0(0)	4(6)	69(100)
Not Detected	151(48)	45(14)	31(10)	10(3)	16(5)	17(5)	14(4)	6(2)	9(3)	18(6)	317(100)
Not tested	101(47)	29(13)	17(8)	5(2)	14(7)	14(7)	13(6)	7(3)	5(2)	10(5)	215(100)
Total	294(49)	86(14)	51(8)	15(2)	30(5)	32(5)	28(5)	19(3)	14(2)	32(5)	601(100)

*601 cases with successful karyotype data represented in Table S3. Excluded data includes cases with failed karyotypes and no karyotype testing done (253/854 cases).
